# Are All Remote Associates Tests Equal? An Overview of the Remote Associates Test in Different Languages

**DOI:** 10.3389/fpsyg.2020.01125

**Published:** 2020-06-30

**Authors:** Jan Philipp Behrens, Ana-Maria Olteţeanu

**Affiliations:** Cognitive Systems Group, Human-Centered Computing, Freie Universität Berlin, Berlin, Germany

**Keywords:** remote associates test, RAT, CRA, creativity, creativity evaluation and metrics, creativity test

## Abstract

The Remote Associates Test (RAT, CRA) is a classic creativity test used to measure creativity as a function of associative ability. The RAT has been administered in various different languages. Nonetheless, because of how embedded in language the test is, only a few items are directly translatable, and most of the time, the RAT is created a new in each language. This process of manual (and in two cases, computational) creation of RAT items is guided by the researchers' understanding of the task. This paper focuses on the question of whether RAT datasets administered in different languages within the literature are comparable. To answer this question, datasets acquired using different RAT stimuli are analyzed qualitatively and quantitatively. Kruskal-Wallis tests are conducted to find out whether there is a significant difference between any of the datasets for a given time frame. Pairwise Mann-Whitney *post-hoc* tests are then used to find out which pairs are different. Significant differences are observed between 18 dataset pairings regarding Accuracy and between 16 in terms of Response Time. The potential sources of these differences are discussed, together with what this means for creativity psychometrics and computational vs. manual creation of stimuli.

## 1. Introduction

The Remote Associates Test is a creativity test that is often used in the literature (Mednick and Mednick, 1971; Ansburg and Hill, 2003; Ward et al., 2008; Cai et al., 2009; Cunningham et al., 2009). A RAT problem given to a participant contains three words, for example, Fish, Mine, Rush; the participant has to come up with a fourth word related to all of the three given words. In this case, Gold is an answer, because the compounds Goldfish, Gold Mine, Gold Rush can be built with it. For a human or a machine (Olteţeanu and Falomir, 2015) to solve the RAT, knowledge about the compound words of a language is needed.

Because solving the RAT relies on knowing various expressions and compound words from a language, native speakers have an advantage and are generally the target population when deploying the RAT. This gives rise to a need for different RAT stimulus sets in different languages.

As the RAT relies on knowledge and expressions that are language-specific, the RAT is, in most part, not translatable between languages. Exceptions to this are the rare cases in which all compounds required as knowledge by a RAT item in a specific language also exist in another language—for example, Goldfisch, Goldmine, Goldrausch as the German counterpart of the above-mentioned query.

As only a few items are translatable, RAT sets of items are created anew in each language by researchers. This means that RAT queries are probably impacted by the language itself and quite likely by the preferences and knowledge of compound words of the authors of the stimulus dataset. The Remote Associates Test (RAT) in the native language of the participants is administered in many creativity studies. Results reported in these studies are, therefore, impacted by the quality and difficulty of RAT items in each language. How can this impact be assessed?

No overview exists of human performance in the RAT/CRA in the different languages. Such an overview would help us understand whether significant differences exist between performance on different RAT problem sets in the various languages in which it is employed. If no significant differences exist, this may indicate that results reported for creativity studies that use the RAT in different languages are, indeed, cross-comparable. If a significant difference does exist, however, the comparability of the RAT as a tool across languages may require more nuance and the development of an understanding of the sources of this difference.

This paper sets out to construct an overview of the RAT across eight languages and two types of RAT (compound and functional) and to provide an initial comparative analysis between RAT sets across all of these languages. Section 2 introduces the different language datasets that will be used. The third section compares the RAT datasets quantitatively and qualitatively. In section 4, results are presented regarding the differences between language and gender. The fifth and last section discusses the results and gives a view of possible future work.

## 2. The Remote Associates Test and languages

Sets of RAT/CRA problems in the following languages were analyzed—please note that some languages have multiple datasets (D):

– German (Landmann et al., 2014)
– Chinese D1 (Shen et al., 2016)
– Chinese D2 (Wu and Chen, 2017)
– Italian (Salvi et al., 2016)
– Romanian (Olteţeanu et al., 2019b)
– Polish (Sobków et al., 2016)
– English D1 (Bowden and Jung-Beeman, 2003)
– English D2 (Olteţeanu et al., 2017)
– English D3 (Olteţeanu et al., 2019a)
– Finnish (Toivainen et al., 2019)
– Russian (Toivainen et al., 2019)

The Dutch (Chermahini et al., 2012) and both of the Japanese versions (Baba, 1982) and (Orita et al., 2018) had to be excluded because either the author was unreachable or the requested data were not sent to us in time.

## 3. Remote Associates Test comparison

A qualitative and quantitative comparison of the above-mentioned RAT datasets is provided in the next sections.

### 3.1. Qualitative Comparison

English datasets D2 and D3 contain different types of items: *compound* vs. *functional*. For compound items, the relationship between the three given words and the answer word is a relationship manifested in language—for example, Gold Fish, Gold Mine, and Gold Rush are compounds that all appear in language. By contrast, the relationship between functional query words and the answer reflects a functional relationship between these words, and there may or may not be a compound linguistic relationship. For example, the relationship between Clockwise and Right or Wrong and Right is a functional relationship. Of the above datasets, English D3 is functional.

Independent of the compound/functional classification, RAT problems have also been divided into two types based on the order of the words: *homogeneous* and *heterogeneous* items. RAT items are homogeneous if the solution word is either a prefix or a suffix to all three of the words in the problem (like in the query Fish, Mine, Rush, where Gold acts as a prefix to each of the query items). Problems are heterogeneous if the solution word is the prefix for some of the words and the suffix to other words in the problem (e.g., in the query River, Note, Account, the answer Bank is a suffix for the first word and a prefix for the other two).

Of the above datasets, the German, Italian, and English D1 distinguish between heterogeneous and homogeneous queries. ANOVAs with task type as a factor were run by the dataset authors on these sets. The task-type factor showed no significant effect on accuracy (the number of queries solved by the participants). Only in the German version was a significant effect of the task-type factor on reaction times observed.

Because of the linguistic differences between Chinese and English, the Chinese authors came up with a character pairing method rather than compound words. In the authors' example, 生(to generate), 天(the sky), and 溫(warm) paired with the solution creates three actual two-character words. The answer, in this case, would be氣(air), and the resulting two-character words are生氣(anger),天氣(weather), and氣溫(temperature).

The Chinese D2 distinguished not between *heterogenous* and *homogeneous* but between *heteronym* and *non-heteronym* words. A heteronym is a word that has the same spelling but different pronunciation and meaning, for example, desert (arid region)/desert (leave). They found that the pass rate on heteronymous items was lower for the 20 and 30-s time limit condition but that the response time was not, indicating that heteronymous items were more difficult.

#### 3.1.1. Test Item Creation

In the Italian study, 150 CRA items inspired by Mednick (1962) were initially tested and then reduced to 122 items by filtering out items that were always or never solved. At the beginning, the German study also contained 150 items. Its creation was based on the original of Bowden and Jung-Beeman (2003) and was later filtered down to 130 items because 13 items had multiple solutions and 7 contained unclear words. The approach of the Romanian study was to first translate items from Bowden and Jung-Beeman (2003) and Salvi et al. (2016). If the translation was impossible (most items), the item was adapted or a single translated word out of the item was used as a seed for the creation of a new item. Afterward, the 198 created items were rated by the authors and five student volunteers in terms of how suitable they were, and then the dataset was reduced to the 111 most suitable items. The Polish dataset was created based on the original items of Bowden and Jung-Beeman (2003) and first contained 50 triads. These were then further reduced to 25 with diverse difficulty and one dominating solution. A subsequent test resulted in another reduction to 17 triads because of low factor loadings. The 47 Finnish items were all created by the research team, whereas the 48 Russian ones contained 12 created items and 36 items adopted from Druzhinin (1999). The authors of the Chinese D1 selected, according to Sio and Rudowicz (2007), 192 out of 288 items previously constructed by Jen et al. (2004), with the criterion that no solutions were repeated or used as problem words. After another reduction based on relative difficulty, the dataset consisted of 128 items. The Chinese D2 authors designed 120 items based on Mednick and Mednick (1971), Bowden and Jung-Beeman (2003), and Jen et al. (2004), of which they finally used the 90 that had a pass rate above 0%.

**Figure 1:**
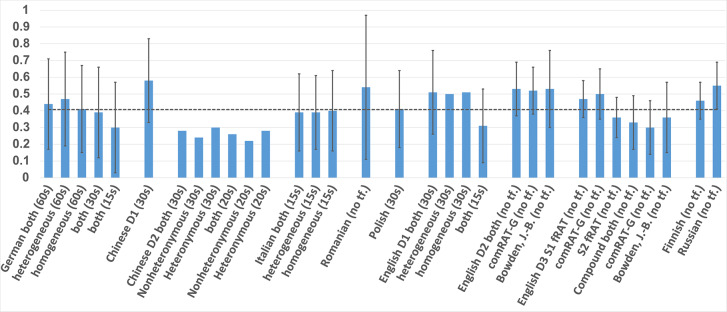
Mean and SD Accuracy of RAT datasets in the different languages.

**Figure 2:**
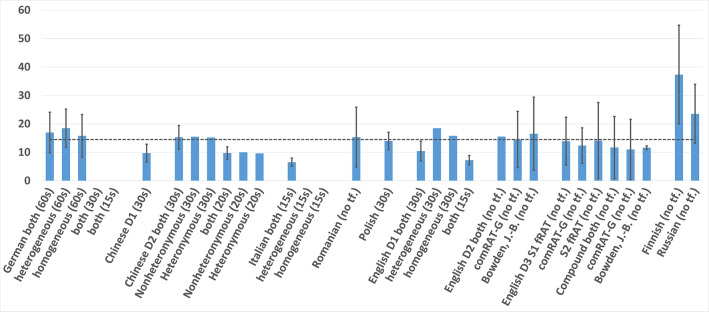
Mean and SD Response Time (in seconds) of RAT datasets in the different languages.

Of the dataset items above, most are manually created. Exceptions to this are items from the English D2 and English D3 datasets. For English D2, (Olteţeanu et al., 2017) successfully attempted the computational creation of RAT items and compared the results with an existing (English D1) normative dataset. For English D3, (Olteţeanu et al., 2019a) applied a computational approach using a new type of language knowledge for the creation of functional items, thus resurrecting an older idea of Worthen and Clark (1971) regarding the existence of such items and their differences from compound items. These items are compared to compound items of a subset of English D1 in the paper. This subset—specifically 24 items from English D1—is marked as Bowden, J.-B. in Figures 1, 2.

### 3.2. Quantitative Comparison

In the following, a descriptive statistics overview of the different datasets is provided.

#### 3.2.1. Descriptive Data

The various RAT datasets contained varying numbers of items, between 17 (Polish) and 144 (English D1). An exception is comRAT-G, which computationally creates 13.4 m items and the frequency-based probabilities of solving them. Furthermore, the various items were deployed either (a) by giving participants different time frames to solve each query, between 2 and 60 s or (b) without setting a time limit. Since 2, 5, 7, 20, and 60-s time frames were only used once across these datasets, only items with a 15 or 30-s time frame or no time frame are analyzed in this paper. Assuming that different solving strategies may be deployed for different time frames, we did not want to average across time frames. The stimuli were deployed on populations of various sizes, with *n* ranging between 26 participants in the English D3 S1 and 317 in the Italian dataset.

As shown in Table 1, Figures 1, 2, the easiest sets to solve were the Chinese D1, with 0.58 accuracy, and the Italian, with a response time of only 6.52 s. The hardest sets seem to be the Chinese D2, with an average accuracy of 0.26 within a 20-s time frame, and the Finnish dataset in terms of response times, with a mean of 37.34 s. The response times of the Russian RAT were also noticeably higher that for the rest (23.53 s). Please note that means and standard deviations were calculated for this paper from the given data where they were not provided by the initial dataset authors.

**Table 1:** Number of elements (|*x*|), sample size (n), mean ($\bar{x}$), and standard deviation (s) of accuracy and response time and Cronbach's α for the RAT in different languages.

	**Time**			**Accuracy**				**RT [s]**		**Cron-**	
	**Frame**			**Sum**		**%**		**Per item**		**Bach's** **α**	
**Language**	**in s**	**|*x*|**	**n**	**$\bar{x}$**	**s**	**$\bar{x}$**	**s**	**$\bar{x}$**	**s**	**Acc**.	**RT**
German both	60	130	80	54.99	34.97	44	27	16.97	7.12	—	—
heterogeneous	60	56	80	26.10	15.79	47	28	18.50	6.70	—	—
homogeneous	60	74	80	30.19	19.17	41	26	15.80	7.50	—	—
German both	30	130	80	—	—	39	27	—	—	—	—
German both	15	130	80	—	—	30	27	—	—	—	—
Chinese D1	30	128	123	74.46	—	58	25	9.74	3.13	0.92	—
Chinese D2 both	30	90	71	25.26	—	28	—	15.31	4.14	—	—
Non-heteronymous	30	60	71	18.07	—	24	—	15.49	—	—	—
Heteronymous	30	30	71	7.19	—	30	—	15.21	—	—	—
Chinese D2 both	20	90	93	23.45	—	26	—	9.77	2.17	—	—
Non-heteronymous	20	60	93	16.76	—	22	—	10.01	—	—	—
Heteronymous	20	30	93	6.69	—	28	—	9.65	—	—	—
Italian both	15	122	317	47.58	28.06	39	23	6.52	1.46	—	—
Heterogeneous	15	66	317	25.48	14.72	39	22	—	—	—	—
Homogeneous	15	56	317	22.12	13.44	40	24	—	—	—	—
Romanian	None	111	63	59.94	47.73	54	43	15.37	10.53	0.93	0.97
Polish	30	17	206	6.90	3.90	41	23	14.02	3.06	0.79	—
English D1 both	30	144	289	72.72	—	51	25	10.45	3.47	—	—
Heterogeneous	30	59	289	29.74	—	50	—	—	—	—	—
Homogeneous	30	85	289	42.93	—	51	—	—	—	—	—
English D1 both	15	144	289	—	—	31	22	7.26	1.65	—	—
English D2 both	None	100	113	52.64	16.16	53	16	—	—	0.94	0.99
comRAT-G	None	50	113	26.20	7.03	52	14	14.52	9.89	0.85	0.99
Bowden, J.-B.	None	50	113	26.41	11.24	53	23	16.56	12.84	0.93	0.99
English D3 S1 fRAT	None	75	26	35.27	7.99	47	11	13.91	8.42	—	—
comRAT-G	None	50	26	25.02	7.26	50	15	12.38	6.23	—	—
English D3 S2 fRAT	None	48	61	17.10	5.77	36	12	14.14	13.39	0.79	0.90
Compound both	None	48	61	15.85	7.60	33	16	11.68	10.96	0.87	0.96
comRAT-G	None	24	61	7.25	3.72	30	16	11.00	10.62	0.75	0.93
Bowden, J.-B.	None	24	61	8.61	5.06	36	21	11.64	0.65	0.85	0.92
Finnish	None	47	67	21.60	5.30	46	11	37.34	17.36	0.73	—
Russian	None	48	67	26.60	6.90	55	14	23.53	10.38	0.83	—

*S1 and S2 reflect different studies of the same article*.

**Figure 3:**
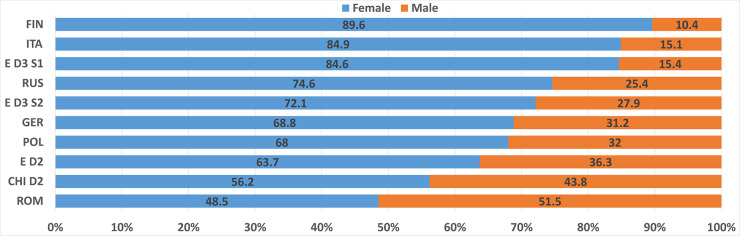
Gender ratio of RAT datasets in the different languages.

The age, level of education, and gender of the participants taking the different RATs also varied, as shown in Tables A1–A5, and Figure 3. For example, 70% of the participants of the Russian RAT were between 20 and 29 years old, whereas over 50% of the English D3S2 were between 30 and 39 years old. The Romanian RAT had the most equal gender ratio, at nearly 50/50, while the Finnish had the worst, with 90% females. Table 1 gives an overview of all of the datasets and various descriptive metrics across all languages.

#### 3.2.2. Cronbach's Alpha

Cronbach's alpha is the most commonly used method for estimating the reliability of a test, as reflected by its internal consistency between items. Scores below 0.5 indicate an unacceptable internal consistency, whereas higher scores indicate a better one. Generally, scores above 0.7 are considered to reflect an acceptable amount of reliability, and an α above 0.9 is excellent. The Cronbach's α scores were calculated by authors for some of the initial papers (see Table 1) and vary between 0.73 and 0.99.

## 4. Results

### 4.1. Language

In order to find out whether differences between results for different languages exist at all, Kruskal-Wallis Tests were conducted for different timesteps and on two existing performance metrics: Accuracy and Response Time. To further investigate which of the language pairings were different, we used pairwise Mann-Whitney tests with Bonferroni-Holm correction as *post-hoc* tests. Heterogeneous and homogeneous items were tested both separately and combined (where possible).

#### 4.1.1. Accuracy

We found a significant effect of group on value for the 30-s time frame [$\chi_{\left(6\right)}^{2}=110.05,$ *p* < 0.0001], the 15-s time frame [$\chi_{\left(2\right)}^{2}=14.58,$ *p* < 0.001], and for no time frame [$\chi_{\left(4\right)}^{2}=18.36,$ *p* < 0.01]. *Post-hoc* tests showed significant differences of means regarding the Accuracy metric for 18 different dataset pairings in different time frames (Table 2). For example, a significant difference exists between Italian vs. German in a 15-s time frame (*p* = 0.00062, α = 0.01667).

**Table 2:** Results of Mann-Whitney testing with Bonferroni-Holm correction regarding the Accuracy.

**Time**	**Dataset pair**		**Holm's method**			
**Frame**			***p***	**Rank**	**α**	**Sig**
15 s	Italian	German	0.0006	3	0.0167	Yes
	Italian	English D1	0.0013	2	0.025	Yes
	German	English D1	0.4694	1	0.05	No
30 s	Chinese D1	Chinese D2	<.0001	21	0.0024	Yes
	Chinese D1	Chinese D2 n.h.	<.0001	20	0.0025	Yes
	Chinese D1	Chinese D2 het.	<.0001	19	0.0026	Yes
	Chinese D2	English D1	<.0001	18	0.0028	Yes
	Chinese D1	German	<.0001	17	0.0029	Yes
	Chinese D2 het.	English D1	<.0001	16	0.0031	Yes
	Chinese D2 n.h.	English D1	<.0001	15	0.0033	Yes
	Chinese D2 het.	Polish	0.0003	14	0.0036	Yes
	Chinese D1	English D1	0.0006	13	0.0038	Yes
	Chinese D2	Polish	0.0006	12	0.0042	Yes
	Chinese D1	Polish	0.0035	11	0.0045	Yes
	Chinese D2 n.h.	Polish	0.0037	10	0.005	Yes
	English D1	German	0.0037	9	0.0056	Yes
	Chinese D2	German	0.0141	8	0.0063	No
	Chinese D2 het.	German	0.0183	7	0.0071	No
	Chinese D2 n.h.	German	0.0889	6	0.0083	No
	English D1	Polish	0.2498	5	0.01	No
	Chinese D2 het.	Chinese D2 n.h.	0.3206	4	0.0125	No
	German	Polish	0.4048	3	0.0167	No
	Chinese D2	Chinese D2 het.	0.4822	2	0.025	No
	Chinese D2	Chinese D2 n.h.	0.6559	1	0.05	No
None	English D3 S2 fRAT	Romanian	<0.0001	10	0.005	Yes
	English D3 S2 fRAT	Russian	0.0006	9	0.0056	Yes
	English D2	English D3 S2 fRAT	0.0054	8	0.0063	Yes
	English D3 S2 fRAT	Finnish	0.0301	7	0.0071	No
	Finnish	Russian	0.0698	6	0.0083	No
	Finnish	Romanian	0.0884	5	0.01	No
	English D2	Finnish	0.3515	4	0.0125	No
	English D2	Russian	0.7583	3	0.0167	No
	Romanian	Russian	0.9033	2	0.025	No
	English D2	Romanian	0.9492	1	0.05	No

#### 4.1.2. Response Time

We found a significant effect of group on value for the 30-s time frame [$\chi_{\left(5\right)}^{2}=158.76,$ *p* < 0.0001] and for no time frame [$\chi_{\left(4\right)}^{2}=64.74,$ *p* < 0.0001]. *Post-hoc* tests using Mann-Whitney tests with Bonferroni-Holm correction showed significant differences of means regarding the Response Time metric for 16 different dataset pairings in different time frames (Table 3). For example, a significant difference was noted between English D2 vs. English D3 S2 fRAT with no time frame (*p* = 0.0095, α = 0.0125).

**Table 3:** Results of Mann-Whitney testing with Bonferroni-Holm correction regarding the RT.

**Time**	**Dataset pair**		**Holm's method**			
**Frame**			***p***	**Rank**	**α**	**Sig**
15 s	English D1	Italian	<0.0001	1	0.05	Yes
30 s	Chinese D2	Chinese D1	<0.0001	15	0.0033	Yes
	Chinese D2	English D1	<0.0001	14	0.0036	Yes
	Chinese D2 n.h.	Chinese D1	<0.0001	13	0.0038	Yes
	Chinese D2 n.h.	English D1	<0.0001	12	0.0042	Yes
	Chinese D2 het.	Chinese D1	<0.0001	11	0.0045	Yes
	Chinese D2 het.	English D1	<0.0001	10	0.005	Yes
	Chinese D1	Polish	<0.0001	9	0.0056	Yes
	English D1	Polish	<0.0001	8	0.0063	Yes
	Chinese D1	English D1	0.1201	7	0.0071	No
	Chinese D2 n.h.	Polish	0.2384	6	0.0083	No
	Chinese D2	Polish	0.2838	5	0.01	No
	Chinese D2 het.	Polish	0.5176	4	0.0125	No
	Chinese D2 het.	Chinese D2 n.h.	0.9018	3	0.0167	No
	Chinese D2	Chinese D2 het.	0.9304	2	0.025	No
	Chinese D2	Chinese D2 n.h.	0.9558	1	0.05	No
None	Finnish	Romanian	<0.0001	10	0.005	Yes
	Finnish	English D3 S2 fRAT	<0.0001	9	0.0056	Yes
	Finnish	English D2	<0.0001	8	0.0063	Yes
	Russian	English D3 S2 fRAT	<0.0001	7	0.0071	Yes
	Russian	Romanian	<0.0001	6	0.0083	Yes
	Finnish	Russian	<0.0001	5	0.01	Yes
	English D2	English D3 S2 fRAT	0.0095	4	0.0125	Yes
	English D3 S2 fRAT	Romanian	0.0701	3	0.0167	No
	English D2	Romanian	0.0749	2	0.025	No
	English D2	Russian	0.1370	1	0.05	No

### 4.2. Gender

In order to measure differences between genders, Welch's unequal variances *t*-test was conducted to measure the difference between means on the two existing performance metrics: Accuracy and Response Time. Moreover, Cohen's d was calculated to measure the effect size.

#### 4.2.1. Differences Between Genders

Significant differences of means for Accuracy with medium effect sizes were observed between genders in:

- Romanian; *t*(59.47) = 2.29^*^, male *M* = 55.25, female *M* = 64.61
- English D3; *t*(24.17) = 2.21^*^, male *M* = 29.78, female *M* = 37.88

as shown in Table A5, but no differences were observed regarding the Response Time. The authors of the Chinese D2 and an older Italian version (Salvi et al., 2015) also stated that gender was not a factor in their experiments.

## 5. Discussion and Further Work

This paper set out to compare the RAT in different languages and across different datasets. Significant differences were observed between multiple languages and datasets on both the Accuracy and Response Time performance metrics.

The significant difference observed between the English D2 and English D3 sets may have as a source the difference between types of items (compound vs. functional).

In the cases in which a significant difference exists between different language datasets, the main potential causes are:

- different population samples are more creative (or at least better at the associative factor in creativity),
- the RAT is more difficult in some languages because of the language itself and the cognitive factors resulting from encoding linguistic knowledge and solving the RAT in that language, and/or
- sets of RAT queries vary in difficulty because they are created without using standardized methods and thus depend on the inspiration and knowledge base of the researchers creating them, or
- the lack of a common time frame.

Other causes could be, as pointed out by our reviewers, differences in the instructions/explanation of the task, in participants' motivations, in the study setting (e.g., fMRI scanner/EEG, etc.), in other tasks performed during the same session, and in whether solution feedback was given, and, also, the associations between the items themselves might affect the difficulty (Luft et al., 2018).

This initial investigation shows that differences between results obtained with the RAT in different languages need to be addressed in more detail. Before cross-comparison of creativity results can be performed, the source of these differences needs to be found. Experimental or analytical setups need to be designed in order to establish which one of the above-mentioned causes, or what combination thereof, is the source of the differences.

An initial thought on establishing comparability could be to attempt to find items that are translatable across the various languages. By keeping stimulus items constant, differences in creativity pertaining to the population or use of language could be established.

However, even if translatable, the same RAT items may not be of the same difficulty in different languages. Some light is shed on this by computational models like comRAT-C (Olteţeanu and Falomir, 2015), essentially models of memory search, which can solve the RAT by organizing their knowledge in a semantic net-like structure, propagating activation through word associations and convergence. comRAT-C's probability of solving a query correlates with human performance. Such models indicate that, even if different RAT queries can be translated in different languages, equivalence does not necessarily exist between them: the number of word associates and the strength of association may not be the same in different languages. Different tools may thus need to be used to try to establish query equivalence.

A potential solution may be to establish a stronger item equivalence in computational terms: for example by using computational RAT query generators like comRAT-G (Olteţeanu et al., 2017) to create sets of items where a high degree of control can be maintained over the number of associates and the association strength of the query words. Such approaches have already proven fruitful in the deployment of more precise empirical designs (Olteţeanu and Schultheis, 2017) and in the creation of other types of items (Olteţeanu et al., 2019a). We have not yet attempted to generate comparable RAT stimulus sets in different languages. To apply the computational approach above for RAT generation in multiple languages would require initial sets of word associations or n-grams for each of the respective languages together with data on how often the n-grams occur within a specific dataset or the frequency of eliciting a particular associate (if a certain number of participants is asked to produce associates).

Another direction of future work would be to establish a creative association measure that transcends the constraints of language such as a visual Remote Associates Test—some work in this direction has already been done by Olteţeanu et al. (2015) and Toivainen et al. (2019). As one of our reviewers very interestingly points out, visual information, though not as varied as language, nonetheless varies in different cultures. The visual RATs would thus not be completely immune to differences, for example, when apple trees are more common in some parts of the world and mango trees in others (our reviewer's example) or when certain objects are more likely to exist, be used, or be central in various cultures. However, these difference may be smaller than linguistic differences for specific sets of objects, and the visual RAT may thus provide a measure with stronger comparability across languages.

This paper gives an overview of RAT datasets in multiple languages and shows that cross-linguistic comparability should not be taken for granted in the case of this broadly used creativity test.

## Data Availability Statement

The original contributions presented in the study are included in the article/supplementary material, further inquiries can be directed to the corresponding author/s.

## Author Contributions

A-MO contributed the conception and design of the study and wrote the first draft of the manuscript. JB performed the statistical analysis. JB and A-MO wrote sections of the manuscript. All authors contributed to manuscript revision and read and approved the submitted version.

## Conflict of Interest

The authors declare that the research was conducted in the absence of any commercial or financial relationships that could be construed as a potential conflict of interest.

## Figures and Tables

**Table A1:** Mean, standard deviation, and range of participant age.

	**ER**	**CHI_***D*2**_**	**ITA**	**POL**	**ROM**
M_*age*_	23.41	22.67	25.30	25.10	—
SD_*age*_	2.93	3.24	8.30	7.60	—
range_*age*_	20–31	18–34	16–65	18–55	18–70

**Table A2:** Percentage of participants in certain age ranges.

	**ROM**	**E_***D*2**_**	**E_***D*3*S*1**_**	**E_***D*3*S*2**_**	**FIN**	**RUS**
<20	4.8	1.8	0.0	2.0	0.0	4.5
20–29	42.9	29.2	11.5	23.0	22.4	70.1
30–39	30.1	31.9	53.8	21.0	23.9	22.4
40–49	14.3	15.0	11.5	20.0	28.3	1.5
50–59	6.3	17.7	23.1	25.0	20.9	1.5
59 <	1.6	4.4	0.0	10.0	4.5	0.0

**Table A3:** Percentage of participants with a certain level of education.

	**ROM**	**E_***D*2**_**	**E_***D*3*S*1**_**	**E_***D*3*S*2**_**
Secondary school	0.0	6.2	11.5	5.0
High school diploma	14.3	23.9	26.9	25.0
Enrolled in undergraduate courses	17.5	17.7	15.4	7.0
Completed undergraduate courses	50.8	30.1	23.1	52.0
Enrolled in postgraduate courses	4.8	5.3	11.5	2.0
Completed postgraduate courses	12.7	16.8	11.5	10.0

**Table A4:** Percentage of participants with certain gender.

	**GER**	**CHI_***D*2**_**	**ITA**	**POL**	**ROM**	**E_***D*2**_**	**E_***D*3*S*1**_**	**E_***D*3*S*2**_**	**FIN**	**RUS**
Female	68.8	56.2	84.9	68.0	48.5	63.7	84.6	72.1	89.6	74.6
Male	31.2	43.8	15.1	32.0	51.5	36.3	15.4	27.9	10.4	25.4

**Table A5:** Welch test results for accuracy without a time frame between genders.

	**ROM female**				**ENG D3 S2 fRAT female**			
	***t***	**df**	***p***	**d**	***t***	**df**	***p***	**d**
ROM male	2.29	59.47	0.03	0.58	—	—	—	—
ENG D3 S2 fRAT male	—	—	—	—	2.21	24.17	0.04	0.70
